# An Alzheimer’s Curriculum to Educate the Current and Future Public Health Workforce

**DOI:** 10.1093/geroni/igaa057.030

**Published:** 2020-12-16

**Authors:** Taylor Kennedy, Molly French, Linelle Blais, Nia Reed

**Affiliations:** 1 Emory University, Atlanta, Georgia, United States; 2 Alzheimer's Association, Washington, District of Columbia, United States; 3 Centers for Disease Control and Prevention, Atlanta, Georgia, United States

## Abstract

Alzheimer’s disease is the 6th leading cause of death among adults in the United States and the 5th leading cause for those aged 65 and older. Nearly 14 million Americans will be diagnosed with Alzheimer’s dementia by 2060, but the public health workforce is struggling to meet current demands. As the older adult population continues to grow, the public health sector will need to ensure a sizable and competent workforce is prepared to meet the needs of those living with dementia as well as their caregivers. In support of national efforts to promote and ensure a competent workforce, the Alzheimer’s Association, Centers for Disease Control and Prevention, and Emory University developed “A Public Health Approach to Alzheimer’s and Other Dementias” (ADOD) curriculum. The free, introductory curricular resource was first piloted by faculty and students at undergraduate schools of public health across the country; however, due to its broad applicability the curriculum has since been updated and expanded to educate graduate students in schools of public health, students in related disciplines, and practicing public health professionals. The curriculum provides an introduction to ADOD as a public health crisis, basics of dementia, the role of public health in addressing the epidemic, and the creation of dementia-friendly communities. The purpose of the curriculum is to educate future public health workforces about ADOD; encourage the current public health workforce to apply knowledge to practice; and seek to improve health outcomes for those living with dementia, as well as their caregivers.

